# Epigenetic Modification of CFTR in Head and Neck Cancer

**DOI:** 10.3390/jcm9030734

**Published:** 2020-03-09

**Authors:** Yonghwan Shin, Minkyoung Kim, Jonghwa Won, Junchul Kim, Seog Bae Oh, Jong-Ho Lee, Kyungpyo Park

**Affiliations:** 1 Department of Physiology, School of Dentistry, Seoul National University and Dental Research Institute, Seoul 110-749, Korea; shinyh81@gmail.com (Y.S.); gloriakim@snu.ac.kr (M.K.); jckim1@snu.ac.kr (J.K.); 2 Department of Neurobiology and Physiology, School of Dentistry, Seoul National University and Dental Research Institute, Seoul 110-749, Korea; caramelapple@snu.ac.kr (J.W.); odolbae@snu.ac.kr (S.B.O.); 3 Department of Oral and Maxillofacial Surgery, School of Dentistry, Seoul National University, Seoul 110-749, Korea; leejongh@snu.ac.kr

**Keywords:** CFTR, head and neck cancer, 5-aza-2′-deoxycytidine, epigenetic modification, DNA methylation, hypermethylation, hypomethylation

## Abstract

Cystic fibrosis transmembrane conductance regulator (CFTR), a cyclic AMP (cAMP)-regulated chloride channel, is critical for secretion and absorption across diverse epithelia. Mutations or absence of CFTR result in pathogeneses, including cancer. While CFTR has been proposed as a tumor suppressing gene in tumors of the intestine, lung, and breast cancers, its effects in head and neck cancer (HNC) have yet to be investigated. This study aimed to define expression patterns and epigenetic modifications of CFTR in HNC. CFTR was expressed in normal but not in HNC cells and tissues. Treatment with 5-aza-2′-deoxycytidine (5-Aza-CdR) was associated with rescued expression of CFTR, whose function was confirmed by patch clamp technique. Further experiments demonstrated that CFTR CpG islands were hypermethylated in cancer cells and tissues and hypomethylated in normal cells and tissue. Our results suggest that CFTR epigenetic modifications are critical in both down-regulation and up-regulation of CFTR expression in HNC and normal cells respectively. We then investigated the impact of CFTR on expressions and functions of cancer-related genes. CFTR silencing was closely associated with changes to other cancer-related genes, suppressing apoptosis while enhancing proliferation, cell motility, and invasion in HNC. Our findings demonstrate that hypermethylation of CFTR CpG islands and CFTR deficiency is closely related to HNC.

## 1. Introduction

Epigenetic modifications occur in both normal developmental and human disease processes [1]. Epigenetic mechanisms, mainly DNA methylation and histone modification, regulate the gene expression of heritable information without changing the DNA sequence [2]. DNA methylation patterns observed in cancer are categorized into hypomethylation or hypermethylation. Specifically, hypomethylation of proto-oncogenes at transcription regulatory regions and hypermethylation of tumor suppressor genes at CpG islands in the gene promoter region [3]. Hypermethylation of CpG islands generally modulates global and site-specific gene expression by altering the level of chromatin condensation to modify accessibility of transcription factors for binding to target genes [4]. While CpG islands are hypomethylated in normal tissue, CpG island hypermethylation localized in the promotor of tumor suppressing genes can cause transcriptional silencing that results in carcinogenesis and tumor progression [5]. This hypermethylation is usually carried out by DNA methyltransferases (DNMTs), such as Dnmt1, Dnmt3a, and Dnmt3b [6]. The well-established epigenetic drug, 5-aza-2′-deoxycytidine (5-Aza-CdR), is incorporated into DNA and inhibits DNMTs and DNA methylation [7] and has been found to restore gene expression associated with the demethylation of CpG islands of genes silenced by hypermethylation [8]. It has been used clinically to treat acute myeloid leukemia and myelodysplastic syndromes [8]. Besides, low dose inhibitors of DNMT and histone deacetylase (HDAC) has been clinically used to treat advanced solid tumors, such as in lung cancer [9] and breast cancer [10], but clinical studies on solid tumors are in process [11].

Head and neck cancer (HNC) is the sixth most frequently diagnosed cancer worldwide. Despite extensive study of HNC, five-year survival rates are relatively low, at approximately 50% of HNC patient, with more than 650,000 HNC cases and 330,000 deaths occur each year [12]. Hence, the new biomarker-guided discoveries and therapeutic targeting is urgently required. While epigenetic modulation has been studied in a few cancers, epigenetic regulation of cystic fibrosis transmembrane conductance regulator (CFTR) in HNC has yet to be reported.

The present study explored the expression patterns of CFTR in head and neck cancer and in normal cells and tissues. Data showed differential expression patterns for CFTR between normal and cancer cells and tissues. We also tested the expression levels and methylation statuses of CFTR in HNC tissues. Then 5-Aza-CdR was used to determine how DNA methylation affected CFTR expression in HNC cells. Similar results were obtained from human tissues wherein CFTR expression was silenced, and those wherein the CFTR CpG islands was controlled by epigenetic modification. We thereby suggest that the balance of DNA methylation in CFTR CpG islands regulates CFTR expression. We further demonstrated the effect of CFTR on HNC using immunostaining, flow cytometry, real-time PCR, CCK-8 assay, caspase3/7 assay, EdU assay, and Matrigel invasion assay. CFTR induction by 5-Aza-CdR treatment attenuated cancer characteristics while CFTR silencing promoted it via tumor invasion, anti-apoptosis, rapid growth, and high cell motility, indicating CFTR is significant in 5-Aza-CdR-induced anti-tumor activity in HNC. CFTR silencing also altered mRNA expression levels of other tumor-related genes. Taken together, these novel findings strongly suggest CFTR could be an effective tumor suppressor in HNC.

## 2. Experimental Section

### 2.1. Cell Culture

HSG cells isolated from a human submandibular gland were used as controls [13]. Human head and neck cancer A253 cells derived from a human submandibular gland tumor were used throughout the study, as were SGT cells derived from human submaxillary gland adenocarcinoma [14]. Cells were cultured in Dulbecco’s modified Eagle’s medium (DMEM) or McCoy’s 5A Medium supplemented with 10% fetal bovine serum, 1% penicillin, and streptomycin and incubated at 37 °C in a humidified atmosphere of 95% O_2_ and 5% CO_2_.

### 2.2. Human Tissue and Ethical Approval

Human head and neck cancer tissues were excised from four adult patients with malignant neoplasms by surgical resection and verified under a microscope. Before surgery, malignant tumors were confirmed from PET-CT and the pathology report. As for the method of harvesting, the most infiltrative part of mass was taken from the excised tumor during surgery as cancer sample, and the tissue taken from the safety margin, at least 1 cm apart from the tumor edge, was used as normal sample. These control and tumor tissue samples were collected and confirmed as non-cancerous or cancerous, respectively, by members of the Pathology Department of Seoul National University Dental Hospital. Human tissues were placed in cold HEPES buffer, immediately after surgical removal for experiments. The HEPES-buffered solution contained (in mmol/L) 140 NaCl, 5 KCl, 1 MgCl2, 1 CaCl2, 10 glucose, and 10 Hepes (pH 7.4 with NaOH). All patients gave informed consent to the use of tissue for research purpose. The use of human tissue samples strictly followed all ethical guidelines and consent forms and were approved by the Institutional Review Board of Seoul National University Dental Hospital (CRI11023G).

### 2.3. 5-Aza-CdR Treatment, Reverse Transcriptase (RT)-PCR, and Real-Time PCR

A253 and SGT cells were treated with 10 μM 5-Aza-CdR for 1, 2, 3, and 4 days, and medium containing 5-Aza-CdR was changed daily. Total RNA was isolated from the untreated A253, SGT, and 5-Aza-CdR-treated cells using Trizol (Ambion, Life Technologies, Carlsbad, California. USA) reagent according to the manufacturer’s instructions, and the purity and concentration of the total RNA were measured using a NanoDrop. Total RNA was isolated as previously reported [15]. cDNA was synthesized from total RNA (2 μg) with reverse transcriptase. Primer sequences for PCR amplification of CFTR, cancer-related genes, and GAPDH for endogenous control were as follows: CFTR (RT-PCR): forward 5′-AAGCTGTCAAGCCGTGTTCT-3′ and reverse 5′-CTGCCTTCCGAGTCAGTTTC-3′; GAPDH: forward 5′-GAAGGTGAAGGTCGGAGTC-3′ and reverse 5′-GAAGATGGTGATGGGATTTC-3′; CFTR (real-time PCR): forward 5′-GGAGATGCTCCTGTCTCCTG-3′ and reverse 5′-CCTCTTCGATGCCATTCATT-3′; PTEN: forward 5′-AGTTCCCTCAGCCGTTACCT-3′, and reverse 5′-AGGTTTCCTCTGGTCCT GG T-3′; TJPI: forward 5′-GTCTGCCATTACACGGTCCT-3′, and reverse 5′- GGCTTAAATCCAGGGGAGTC-3′; CDH1: forward 5′-TACACTGCCCAGGAGCCAGA-3′ and reverse 5′-TGGCACCAGTGTCCGGATTA-3′. Previously described primer sequences were also used to study the impact of CFTR on tumor-related genes [16,17,18]. cDNA was applied as a template for CFTR amplification, and RT and real-time PCR conditions were as follows: one cycle at 95 °C for 5 min; 35 cycles of denaturation at 94 °C for 30 s; annealing at 56 °C for 30 s; extension at 72 °C for 30 s; and followed by a final cycle at 72 °C for 10 min. The RT-PCR products were separated on a 1.2% agarose gel via electrophoresis.

### 2.4. Western Blot Analysis

The harvested cells were washed with Phosphate Buffered Saline (PBS) and dissolved in lysis buffer. Protein samples were separated on an 8% SDS-PAGE and transferred to a nitrocellulose membrane. After blocking with 10% non-fat milk (Seoul-milk, Seoul, Korea), the membrane was incubated at 4 °C with primary rabbit CFTR antibody (Santa Cruz Biotechnology, Santa Cruz, CA, USA, 1:1000) and washed with TBST. After washing, the membrane was incubated at room temperature (RT) with an HRP-conjugated secondary antibody (Santa Cruz Biotechnology, 1:5000) for 1 h, then visualized using an ECL reagent (Thermo Fisher Scientific, Waltham, MA, USA).

### 2.5. Immunofluorescence

Cells were grown on cell culture slides and treated with 10 μM 5-Aza-CdR (Sigma-Aldrich, St. Louis, MO, USA) for 3 days. After treatment, cells were fixed in 4% paraformaldehyde and washed with PBS. Cells were then blocked with 10% donkey serum and incubated overnight with anti-human CFTR primary antibody (1:200, Santa Cruz Biotechnology) at 4 °C. After washing with PBS, cells were incubated with Alexa Fluor^®^ 488 donkey anti-mouse IgG secondary antibody (1:200) for 1 h at RT. HSG cells and A253 cells with or without 5-Aza-CdR treatment were mounted with Vectashield H-1200, including DAPI nuclear stain (Vector Laboratories, Cambridgeshire, UK) and visualized on an LSM 700 Laser Confocal Scanning Microscope (Carl Zeiss, Germany).

### 2.6. Whole Cell Patch Clamp Recordings

For whole cell patch clamp recordings, A253 cells exposed to 5-Aza-CdR for 3 consecutive days were detached by 0.05% trypsin treatment and re-plated on poly-D-lysine-coated coverslips. Recordings were made 2 h after re-plating. To isolate Cl^-^ current, Na^+^ and K^+^ were excluded from the pipette and external solutions. The pipette solution for voltage clamp recordings contained (in mmol/L): 140 NMDG, 1 MgCl_2_, 1 Mg ATP, 10 HEPES, and 0.5 EGTA, adjusted to pH 7.2 with HCl. The external solution contained (in mmol/L): 140 NMDG, 1.2 MgCl_2_, 1.2 CaCl_2_, 10 HEPES, and 10 D-glucose, adjusted to pH 7.2 with HCl [19,20]. Whole cell current responses were recorded with an Axopatch 200B amplifier and Digidata 1322A (Molecular Devices CA, USA), filtered at 1–2 kHz and sampled at 5 kHz. Pipette resistance was 3–4 MΩ. 250 mM 8-Bromo-cAMP (Tocris, Bristol, UK) in distilled water was diluted to a final concentration of 200 μM. 10 mM CFTR_inh_-172 (Sigma–Aldrich) in DMSO was diluted to a final concentration of 10 μM. The external solution was gravity fed at a rate of 1.5–2 mL/min. All chemicals for preparing the above solutions were purchased from Sigma–Aldrich unless otherwise indicated.

### 2.7. Methylation-Specific PCR

Genomic DNA was isolated using the QIAamp DNA Blood Mini Kit (Qiagen, Valencia, CA, USA) from HSG, A253, and SGT cells (before and after treatment with 5-Aza-CdR), and from HNC and normal tissues. For methylation-specific PCR (MSP), bisulfite modification of genomic DNA was performed with the EpiTect Bisulfite Kit (Qiagen) as described previously [15]. MSP was performed with bisulfite-treated genomic DNA using specific primers for methylated or unmethylated forms of the CFTR CpG islands. The following methylation-specific primer sequences were used: M forward 5′-TATATTGTCGCGGAATTTTTC-3′ and M reverse 5′-TTTCCCGATAATCCTAATCG-3′; U forward 5′-ATTTATATTGTTGTGGAATTTTTT-3′ and U reverse 5′-CCTTTTCCCAATAATCCTAATCA-3′.

### 2.8. Bisulfite Sequencing

Genomic DNA (2 μg) was modified by sodium bisulfite conversion reaction with the EpiTect Bisulfite Kit (Qiagen) according to the manufacturer’s instructions. The modified genomic DNA was amplified with bisulfite primer sequences of the CFTR CpG islands designed by Methyl primer express software as follows: forward 5′- TTGGGTTAAAAAGGATAGATAAGG -3′ and reverse 5′- AAAAACTTCCTAAACCCTCCTT -3′. The PCR reactions were carried out as follows: one cycle at 94 °C for 5 min; 35 cycles of 94 °C for 45 s and 55 °C for 45 s; and a final cycle of 72 °C for 45 s at the end. The PCR products were purified with a Gel Extraction Kit (Qiagen) and ligated into the pGEM-T easy vector (Promega, Madison, WI, USA). Five separate clones, each from HSG cells, A253 cells, and SGT cells (before and after treatment with 5-Aza-CdR) were selected for bisulfite sequencing analysis according to previously described methods [15].

### 2.9. GEO Dataset Analysis and PPI network (STRING)

Human methylation profiling arrays for HNC and normal adjacent tissues were downloaded from the NCBI Gene Expression Omnibus (GEO, http://www.ncbi.nlm.nih.gov/geo/) [21]. The methylation dataset GSE25093 consisted of 18 normal and 91 HNC tissue samples [21,22]. CpG-targeting probe IDs were annotated with corresponding genes, and the methylation levels of selected genes were collected. The protein-protein interaction (PPI) network is generated by STRING v11.0 database, a search tool for retrieval of functional associations between genes and proteins (http://string-db.org/).

### 2.10. Small Interfering RNA Transfection

A253 cells were transfected at 70–80% confluence with ON-TARGET plus SMART pool siRNA against CFTR (Dharmacon, Boulder, CO, USA) or scrambled siRNA (Non-targeting pool D-001810; Dharmacon) using DharnaFECT 1 siRNA Transfection Reagent according to the manufacturer’s instructions. The corresponding target sequences were 5′-GAACACAUACCUUCGAUAU-3′, 5′-GUACAAACAUGGUAUGACU-3′, 5′-GUGAAAGACUUGUGAUUAC-3′, and 5′-GCAGGUGGGAUUCUUAAUA-3′ for CFTR.

Briefly, 50 nM CFTR siRNA, scrambled siRNA, or transfection reagent were incubated respectively with Opti-MEM medium (Gibco Life Technologies, NY, USA) for 5 min. Then, each siRNA was mixed gently with reagent and incubated for 20 min. Finally, whole medium with serum without antibiotic was added to the mixture. The cells were transfected with this medium for 24 h, after which other experiments were conducted.

### 2.11. Proliferation Assay (CCK-8 Assay and EdU Click-iT™ Assay)

Cell proliferation was estimated using the Colorimetric Cell Counting Kit 8 Assay Kits (CCK-8; Dojindo Laboratory, Korea), which consists of WST-8 (2-(2-methoxy-4-nitrophenyl)-3-(4-nitrophenyl)-5-(2,4-disulfophenyl)-2H-tetrazolium and monosodium salt). Cells were incubated on a 96-Cell Culture Microplate and incubated with each treatment for 2.4, 24, 48, and 72 h and growth was then measured as previously described [23]. Briefly, after treatment, the cells were incubated with a mixture of media and CCK-8 solution for 2 h. Proliferation rates were quantified by absorbance at 450 nm, proportional to the number of living cells, using a Synergy 2 microplate reader (BioTek).

After a 3-day treatment, proliferation was visualized with a Click-iT™ EdU assay (Invitrogen/Molecular Probes, Eugene, OR). The presence of 5-ethynyl-2′-deoxyuridine (EdU)-labeled cells from the S phase of the cell cycle indicates cell proliferation. Azides combined EdU and Alexa Fluor^®^ 594 (red) fluorophore for visualization Cells were incubated in the EdU cocktail for 30 min and washed 3 times with PBS. Samples were then permeabilized in 1% Triton 100X for 15 min, washed 3 times with PBS, and fixed in 4% paraformaldehyde for 10 min. After preparing 1× working buffer additive solution, reaction cocktail was made fresh and used within 15 min. Upon completion of the EdU Click-iT™ reaction, nuclei were labeled with Hoechst (Sigma-Aldrich, 33342) diluted to 1:1000 for 50 min, and mounted in Limonene mounting medium onto glass slides and sealed. Results were visualized by confocal microscopy on an LSM700.

### 2.12. Apoptosis Assay (PE/annexin-V and Caspase 3/7 Assays)

Flow cytometry analysis was performed to assess the presence of apoptotic cells. Prepared cells were collected and washed with cold PBS. A total of 1 × 10^6^ cells were resuspended in 100 µL annexin-V binding buffer (1.4 M NaCl, 25 mM CaCl_2_, 0.1 M HEPES, pH 7.4) was added to culture tubes and mixed with 5 µL of PE Annexin-V and 5 µL of 7-AAD (51-65875X, 51-68981 E; BD Biosciences, CA, USA). Each sample was gently vortexed or shaken for 10 min at RT in the dark, and 3 mL binding buffer was added to each tube. Each tube was maintained in the dark and on ice for evaluation with the FACSCalibur flow cytometer (BD Biosciences). Data were analyzed by CELL Quest software (BD Bioscience).

The activity of caspases 3 and 7 was detected with Caspase 3/7 Green Detection Reagent (C10423, Invitrogen). After the activation of caspases 3 and 7 in apoptotic cells, a four amino acid peptides in the reagent is cleaved and releases nucleic acid binding dye that binds to DNA for visualization. After treatment, cells (1 × 10^5^ cells/cm^2^) were plated in black 96-well plates. Cells were then incubated in clear, complete medium with 3 μM caspase3/7 and Hoechst diluted to 1:1000 for 50 min at 37 ℃, and then directly evaluated by LSM 700 with FITC/Alexa Fluor 488 filter settings. Fixation was avoided as the caspase 3/7 reagent can be altered by fixation.

### 2.13. Single Cell Motility Study (Time-Lapse Imaging)

Cells were plated at low confluence (5 × 10^4^ cells/cm^2^) in a 6-well plate after cell preparation and 3 days of treatment. After cell attachment, cell motility was monitored every 30 min for 24 h by a JuLI™ Stage automated cell imaging system (NanoEntek, Seoul, Korea) at a 4× objective. Time-lapse imaging cells were kept at 37 °C in a humidified atmosphere containing 5% CO_2_ to maintain live cells. Thirty cells in each plate were randomly chosen for analysis and tracked using the ImageJ Manual Tracking plug-in (National Institutes of Health, Bethesda, MD, USA). Each experiment was repeated three times. The ImageJ Chemotaxis and Migration tool (Ibidi GmbH, Gräfelfing, Germany) was used to quantitate and visualize data; velocity was represented as accumulated distance (μm) divided by time (min).

### 2.14. Matrigel Invasion Assay

A Matrigel invasion assay was carried out using 24-well Corning Bio Coat Matrigel Invasion Chambers with 8 μm pore polycarbonate membrane inserts (Corning Incorporated, #354480, MA, USA) as described previously [13]. The chambers were pre-coated with Matrigel and rehydrated with serum-free media, and then incubated for 1 h at RT prior to the experiment. After 3-day treatments of prepared cells, cells were seeded into the tops of the upper chambers at 5 × 10^5^ cells/cm^2^. Chemoattractant (20% FBS in DMEM) was placed in the lower chamber. Cells were incubated at 37 °C in a humidified atmosphere containing 5% CO_2_ for 24 h, after which uninvaded cells on the inside of the upper chamber were removed by 3× scrubbing with cotton swabs and 3× washing with PBS. Invaded cells on the lower side of the membrane were fixed in 4% paraformaldehyde for 10 min and then stained with 1% Crystal violet (Sigma-Aldrich) in 2% EtOH for 30 min, followed by five washings with PBS. Remaining cells were washed with water and dried completely. The invaded cells were visualized using a Leica S6D stereo microscope (Leica Microsystems Inc., Bannockburn, IL, USA) at 1.25× magnification. The experiment was independently repeated three times.

### 2.15. Statistical Analysis

All experiments were repeated at least three times. Statistical analysis was conducted using one- or two-way ANOVA on GraphPad Prism 5 software (GraphPad Software, Inc., La Jolla, CA, USA). *p* -values of less than 0.05 were considered statistically significant.

## 3. Results

### 3.1. Silencing and Reactivation of CFTR in A253 Cells

**Figure 1:**
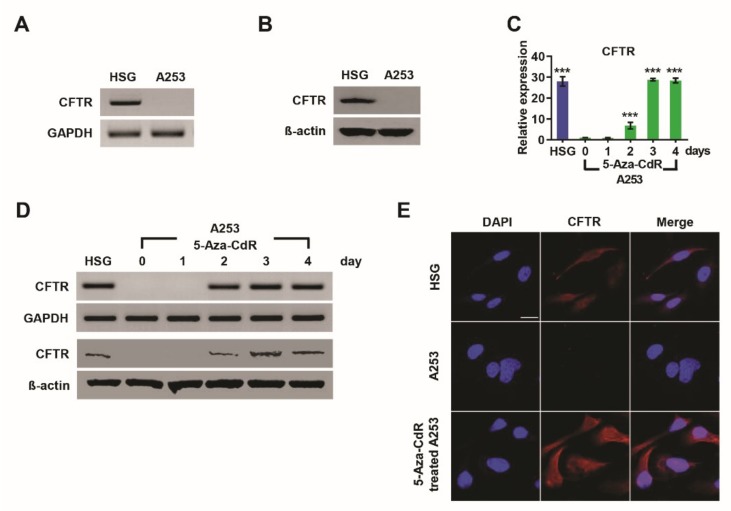
Induction of cystic fibrosis transmembrane conductance regulator (CFTR) expression by 5-aza-2′-deoxycytidine (5-Aza-CdR) in A253 head and neck cancer. CFTR mRNA and protein expression levels were assessed by reverse transcriptase (**A**) (RT)-PCR, (**B**) Western blot and (**C**) real-time PCR. A lack of CFTR expression was observed in A253 cells. Human submandibular gland (HSG) cells were used for comparison. A253 cells were treated with 10 μM 5-Aza-CdR (DNA methyltransferase inhibitor) for 24, 48, 72, or 96 h followed by (**C**) real-time PCR; (**D**) RT-PCR and Western blot (upper and lower band). Data are expressed as mean ± SD. CFTR expression was upregulated by 5-Aza-CdR in a time dependent manner, and maximum expression was reached at 3 days. (**E**) Immunostaining to confirm CFTR expression in HSG, A253, and 5-Aza-CdR-treated A253 at 3 days. Red, CFTR; blue, DAPI nuclear stain. Scale bar = 20 μm. Downregulation of CFTR expression in A253 cells; restoration of CFTR in 5-Aza-CdR-treated A253 cells. All experiments were performed in triplicate. Significance was assessed by one-way ANOVA with Bonferroni’s test. *** *p* < 0.001.

CFTR expression patterns were studied in HSG and A253 cells by RT-PCR. Weak or no expression of CFTR mRNA was observed in A253 cells compared with normal HSG cells (Figure 1A). Western blot was also used to explore CFTR protein expression levels (Figure 1B), and a lack of CFTR protein expression was detected in A253 cells. Epigenetic mechanisms can regulate gene silencing by DNA (hyper)methylation [24]. We explored whether CFTR expression is regulated by DNA methylation in A253 cells using 5-Aza-CdR treatment and control cells were treated with DMSO as a vehicle. CFTR mRNA was reactivated in A253 cells treated with 5-Aza-CdR in a time-dependent manner (Figure 1C,D), and reactivated mRNA CFTR expression was observed after 2 days of treatment with 10 µM 5-Aza-CdR. mRNA expression levels increased significantly over the next 2 to 4 days (Figure 1, upper lane in D). Western blot analysis (Figure 1, third lane in D) also showed increased CFTR protein levels in A253 cells after 5-Aza-CdR treatment in a similarly time-dependent manner. The same pattern was observed in SGT cells (Supplemental Figure S1A,B).

Changes in CFTR protein levels were further explored by immunofluorescence microscopy (Figure 1E), with CFTR localization in HSG cells used as a positive control (Figure 1E). CFTR protein was not detected in A253 cells, but strong protein CFTR expression was detected after 5-Aza-CdR treatment in A253 cells for 3 days. These results suggest that the transcription of CFTR is silenced by hypermethylation but recovered by 5-Aza-CdR-induced demethylation.

### 3.2. Functional Analysis of CFTR in A253 Cells

**Figure 2:**
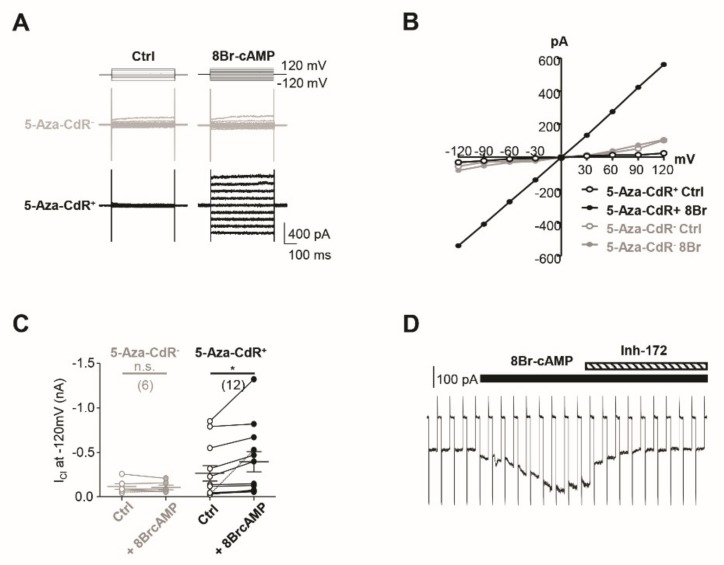
8-Br-cAMP-sensitive chloride current is inhibited by CFTRinh-172 in 5-Aza-CDR-treated A253 cells. (**A**) A253 cells were stimulated with 200 μM 8-Br-cAMP to measure the cAMP-sensitive chloride current. Black and gray traces represent chloride currents recorded in A253 cells with or without 5-Aza-CdR pretreatment, respectively. (**B**) Voltage–current relationship of chloride current before (ctrl, open circle) and after (8-Br-cAMP, closed circle) 8-Br-cAMP treatment of cells in panel (**A**). (**C**) Chloride current amplitude recorded at −120 mV before (ctrl, open circle; *n* = 6; paired Student’s *t*-test, *p* = 0.3907; nonsignificant, n.s.) and after (8-Br-cAMP, closed circle; *n* = 12; paired Student’s *t*-test, *p* = 0.0262; * *p* < 0.05) 8-Br-cAMP treatment in A253 cells. Black and gray trials represent current amplitude from A253 cells with and without 5-Aza-CDR pretreatment, respectively. Trials in panel (**A**) are distinguished by dotted lines. (**D**) Inward chloride current was evoked by −120 mV voltage steps from 0 mV holding potential for 500 ms at 5 s intervals (*n* = 4). 8-Br-cAMP was associated with an increase of inward current, which was suppressed to basal levels by 10 μM CFTR_inh_-172 (Inh-172) treatment.

CFTR-induced chloride currents in HSG cells are well-established in studies using whole-cell patch clamping [25,26]. cAMP-sensitive chloride currents have been observed in HSG, and the CFTR-induced current was nearly abrogated in response to 10 μM CFTR_inh_-172 [25]. In this study, we further confirmed functional CFTR expression in 5-Aza-CdR-treated A253 cells by evaluating cAMP-activated chloride current in these cells via whole cell patch clamp recording. Intracellular cAMP levels were increased by 8-Bromo-cAMP (8-Br-cAMP) treatment (Figure 2A,B), a cell-permeable cAMP analog that induces CFTR currents [27]. Upon 200 μM 8-Br-cAMP treatment, a significant increase in chloride current was observed in the 5-Aza-CdR-treated A253 cells (chloride current at −120 mV, ctrl vs. 8-Br-cAMP; −0.27 ± 0.09 nA vs. −0.40 ± 0.11 nA, Figure 2C). 8-Br-cAMP-sensitive chloride currents in these cells occurred independently of time and responded to voltage steps ranging from −120 mV to 120 mV in an ohmic voltage-current relationship (Figure 2A,B). However, naïve A253 cells were unresponsive to 8-Br-cAMP (chloride current at −120 mV, ctrl vs. 8-Br-cAMP; −0.12 ± 0.03 nA vs. −0.11 ± 0.03 nA, Figure 2A–C). 8-Br-cAMP sensitive currents were suppressed to basal levels in four of the four cells tested after exposure to 10 μM CFTR_inh_-172, a selective CFTR inhibitor, indicating that 8-Br-cAMP-sensitive currents are CFTR currents (Figure 2D).

### 3.3. Methylation Pattern and Status of CFTR in HSG and A253 Cells

**Figure 3:**
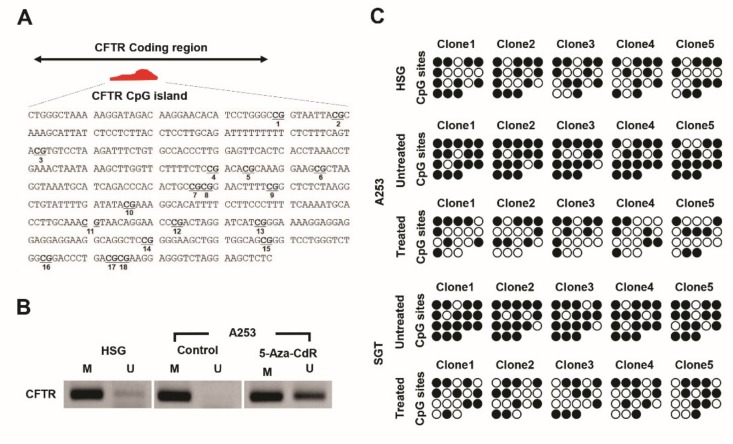
CpG island hypermethylation and hypomethylation of CFTR in A253 cells before and after treatment with 5-Aza-CdR. (**A**) The CFTR CpG islands (GenBank #NM 031506) were analyzed. This coding region consists of 18 CG pairs as potential CpG sites within this sequence. These 18 CG sites are bolded and underlined. (**B**) Methylation levels of HSG and A253 with or without 5-Aza-CdR were assessed using methylation-specific PCR (MSP) in a sodium bisulfate-modified DNA sample. Methylation, M; unmethylation, U. Lack of unmethylated band in A253 cells. Strong unmethylated band in 5-Aza-CdR-treated A253 cells showing hypomethylation of CFTR. (**C**) Bisulfite genomic sequencing showing CpG island methylation of CFTR. (Unmethylated cytosine, open circle; methylated cytosine, closed circle.) Hypermethylation patterns in A253 cells compared with HSG cells. Hypomethylation of CFTR CpG islands in A253 cells in the presence of 5-Aza-CdR. All experiments were performed in triplicate.

Results in Figure 1 and Figure 2 imply that CFTR expression in A253 cells is silenced by DNA methylation of CFTR CpG islands. We used Methyl Primer Express software (Thermo Fisher Scientific) to find a CpG islands in CFTR and identified 18 CGs on this island (Figure 3A). Previously, we reported on total 5-methylcytosine (5-mC) levels to assess global DNA methylation in 5-Aza-CdR-treated or control A253 cells and compared these values with those from HSG cells [28,29]. On this reported basis, we expected total 5-mC levels in HSG and A253 cells to differ. Compared with HSG cells, A253 cells were hypermethylated which is related to CFTR expression silencing in A253 cells.

Methylation patterns on CFTR CpG islands in 5-Aza-CdR-treated or untreated A253 cells were investigated using methylation-specific PCR (MSP). HSG cells were used as controls, and a strong methylated band and the weak unmethylated band were observed in A253 cells (Figure 3B). The strength of the unmethylated band was improved in 5-Aza-CdR-treated A253 cells (Figure 3B). We obtained these same results from SGT cells (Supplemental Figure S1C). These data support our hypothesis that reactivation of CFTR expression by 5-Aza-CdR in A253 cells occurs as a result of epigenetic changes from hypermethylated to hypomethylated status in the CFTR CpG islands.

Bisulfite sequencing of CFTR CpG islands was performed with 5-Aza-CdR treated or untreated A253 cells to assess the DNA methylation status of CFTR. Most of the CFTR CpG sites were methylated in the five A253 and SGT cells (Figure 3C). The CFTR CpG sites of the 5-Aza-CdR-treated A253 cells were considerably less methylated than in the untreated A253 cells (Figure 3C). Analysis of CFTR DNA methylation statuses in 5-Aza-CdR-treated A253 cells (Figure 3C) revealed a similar methylation pattern in HSG cells. We also assessed the methylation statuses of CFTR CpG islands in 5-Aza-CdR-treated or control SGT cells, again with results similar to those in A253 cells. These findings indicate that epigenetic modification, such as hypermethylation and hypomethylation, may be crucial regulatory mechanisms for silencing and reactivation of CFTR in HNC.

### 3.4. Epigenetic Analysis of CFTR CpG Islands in Human Tissue

**Figure 4:**
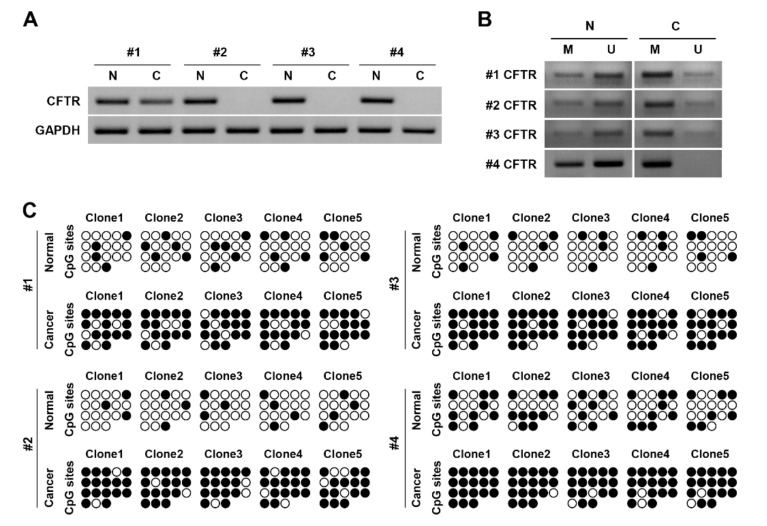
CFTR deficiency and CpG island hypermethylation in HNC patient tissues. (**A**) Total RNA was extracted from four pairs of normal tissues and head and neck cancer tissues, and RT-PCR was used to determine expression levels of CFTR in HNC. CFTR expression was decreased #1 or highly deficient #2–4 in cancer tissues, while CFTR was highly expressed in normal tissues. Cancer tissue, C; normal tissue, N. (**B**) Methylation of CFTR was analyzed by MSP. A CFTR methylated band was strong in cancer tissues while a CFTR unmethylated band was relatively strong in normal tissues (**C**) Bisulfite genomic sequencing of CpG islands of CFTR was performed in cancer and normal tissues. (unmethylated cytosine: open circle, methylated cytosine: closed circle) CpG island hypermethylation of CFTR was apparent in cancer tissue. All experiments were performed in triplicate.

We then studied CFTR expression in four pairs of human cancer tissue samples and found that CFTR was silenced in three out of the four (Figure 4A). Although we did detect a CFTR band in one of these cancer samples, the band was weaker than that in normal tissue, suggesting downregulation of CFTR.

We clarified CFTR methylation status via MSP analysis of CFTR CpG islands. Most normal tissues showed a weak methylated band and strong unmethylated band, whereas most NNC tissues showed a strong methylated band and faint unmethylated band, suggesting hypermethylation of CFTR in HNC tissue (Figure 4B). We then performed bisulfite sequencing of CFTR CpG islands on four normal and cancerous human tissue samples to evaluate their methylation patterns at CFTR CpG islands. Each of the four individual HNC tissue clones showed increased levels of methylated cytosine in cancer tissues compared with normal tissues, indicating that CFTR CpG islands were more methylated in these samples than in normal tissues (Figure 4C), and confirming CpG island hypermethylation-induced CFTR silencing in these cancer tissues.

### 3.5. Epigenetic Regulation of CFTR in Cancer-Related Genes in HNC Cells and DNA Methylation Analyses of Head and Neck Cancer Tissue

**Figure 5:**
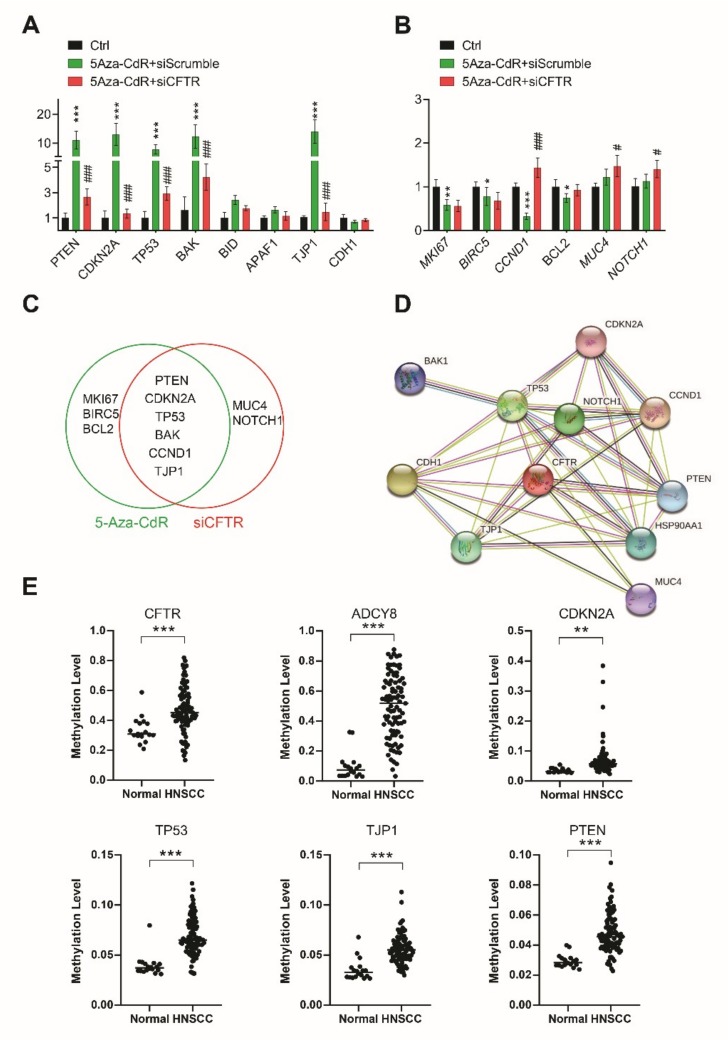
The interaction of CFTR with cancer-related genes; Analysis of DNA methylation profile data from GEO, Gene Expression Omnibus. (**A**-**C**) Real-time PCR of mRNA expression of cancer-related genes with or without 5-Aza-CdR or CFTR siRNA in A253 cells. (**A**) Tumor suppressor genes or anti-cancer genes, such as PTEN, CDKN2A, TJP1, TP53, BAK, BID, APAF1, and CDH1. (**B**) Tumor progression genes or oncogenes, such as MKI67, BIRC5, CCDN1, BCL2, MUC4, and NOTCH1. (**C**) Venn diagram representing the genes significantly changed by both 5-Aza-CdR and siCFTR (overlapping circles, PTEN, CDKN2A, TJP1, TP53, BAK, and CCDN1), 5-Aza-CdR only (green right circle, MKI67, BIRC5, and PCL2), or siCFTR only (red left circle, MUC4 and NOTCH1). All samples were run in triplicate. Data are expressed as mean ± SD (* or ^#^, *p* < 0.05; ** or ^##^, *p* < 0.01; *** or ^###^, *p* < 0.001) using two-way ANOVA with Bonferroni’s test. * denotes significance compared with unstimulated control; # significance compared with 5-Aza-CdR-treated cells. (**D**) The protein-protein interaction (PPI) network of CFTR in the evidence view. Nodes, proteins; links, physical interaction between proteins. (**E**) Comparison of DNA methylation levels of six genes in 18 normal and 91 HNC samples.

To investigate CFTR involvement in HNC, we analyzed the effects of CFTR on other genes and functions involved in HNC. We first assessed whether CFTR modifications affected the expression of other cancer-related genes in HNC. CFTR expression before and after CFTR siRNA treatment was compared with scrambled siRNA in the presence of 5-Aza-CdR by Western blot assay, and data indicated CFTR silencing (Supplemental Figure S2A). We found that CFTR silencing via siRNA inhibited tumor suppressor genes, such as PTEN, CDKN2A, TP53, TJP1, and BAK, while 5-Aza-CdR amplified them (Figure 5A). 5-Aza-CdR interestingly decreased levels of genes that promote tumor progression, such as CCND1, MKI67, BIRC5, and BCL2, but failed to decrease MUC4 and NOTCH1. CFTR silencing enhanced the expression of CCND1, NOTCH1, and MUC4 but not MKI67 (Figure 5B). Figure 5C summarizes significant gene changes associated with 5-Aza-CdR and/or CFTR silencing. The CFTR-dependent tumor-related genes identified perform a range of diverse functions [30]. In our study, among proliferation-related genes, PTEN, CDKN2A, and CCND1 were regulated by CFTR, but MKI67 and BIRC5 were regulated only by 5-Aza-CdR, and not by CFTR. We also found that CFTR silencing occurred in proportion to lowered expression of apoptosis-related genes such as TP53, PTEN, and BAK, whereas BID, BCL2, and APAF1 were not influenced by CFTR. CFTR inhibition reduced transcription of TJP1, NOTCH1, and MUC4, which are related to invasion, cell motility, adhesion, EMT, or pre-metastasis. Our findings strongly suggest that CFTR is a tumor suppressor in HNC. Figure 5D illustrates the protein–protein interaction (PPI) network of 11 cancer-related genes, including CFTR, to represent the predicted functional partners of CFTR that are generated by STRING database. Nodes indicate proteins, and links indicate physical interactions between proteins. Direct (physical) and indirect (functional) interactions with CFTR have been observed. These genes are involved in microRNA cancer, cellular senescence, the cell cycle, tight junctions, responses to infection, cell cycle, focal adhesions, apoptosis, and cellular signaling. Next we analyzed GEO dataset of methylation profiles from 18 normal and 91 HNC human samples (GSE25093). We found concomitant changes in the profiles by assessing the methylation levels of tumor suppressor and CFTR-related genes (Figure 5E). CDNK2A, TP53, TJPI, PTEN, ADCY8, and CFTR genes were highly methylated, and the methylation levels of individual genes encompassed a broad range.

### 3.6. Epigenetic Induction of CFTR Regulates Antiproliferation and Apoptosis

**Figure 6:**
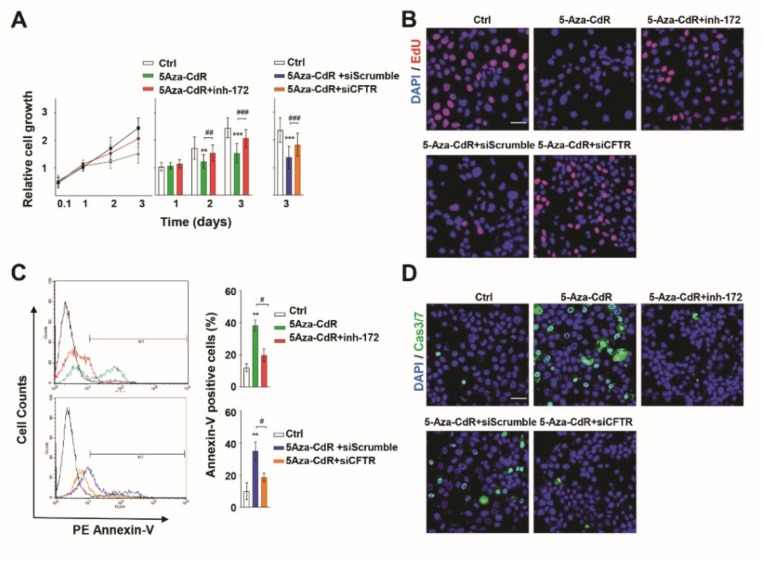
Epigenetic induction of CFTR; proliferation and apoptosis in A253 cells (**A**) Cell growth was measured for 2.4, 24, 48, and 72 h in A253 cells with DMSO (vehicle), 5-Aza-CdR only, or CFTR_inh_-172 + 5-Aza-CdR by CCK-8 assay (left and middle graphs). Cell growth was also compared among control, scrambled siRNA + 5-Aza-CdR, and CFTR siRNA + 5-Aza-CdR (right graph) (*n* = 15). CFTR silencing with CFTR siRNA or CFTR_inh_-172 prevented 5-Aza-CdR effects. Cell growth was inversely proportional to CFTR functional expression. (**B**) Proliferating cells (red) were detected with 5-ethynyl-2′-deoxyuridine (EdU) and compared with total cells by nuclear staining (Hoechst, blue). Scale bar = 50 μm (*n* = 3). Proliferation suppressed by 5-Aza-CdR treatment, increased in CFTR-silenced (CFTR_inh_-172 or CFTR siRNA) + 5-Aza-CdR treatment. (**C**) Apoptotic cells analyzed by PE/annexin-V assay using flow cytometry after 3 days’ treatment (left side) showing 5-Aza-CdR-induced apoptosis and CFTR induced anti-apoptosis. Quantification of annexin-V assay in graphs (right side) of three experiments (*n* = 3). (**D**) Caspase 3/7 activity (Cas3/7, green) was detected by immunostaining assay (*n* = 3). Active caspase 3/7 activation observed in 5-Aza-CdR-treated cells with or without scrambled siRNA but prevented by CFTR inhibition. Nuclei stained with Hoechst (blue). Data are mean ± SD. One and two-way ANOVA with Bonferroni’s multiple comparison tests for significance (* or ^#^, *p* < 0.05; ** or ^##^, *p* < 0.01; *** or ^###^ , *p* < 0.001). * denotes statistical significance compared with control, # denotes significance compared with 5-Aza-CdR-treated cells.

Unchecked cell growth and division are indicators of malignant tumors lacking a tumor suppressor gene [31]. We assessed the cell growth of each group using a CCK-8 assay for 0.1 to 3 days (Figure 6A). At 2–3 days, a significant reduction in cell growth was detected in 5-Aza-CdR-treated A253 cells compared with controls (DMSO). Despite treatment with 5-Aza-CdR, cell growth was increased upon blocking of CFTR activation with CFTR_inh_-172 as compared with 5-Aza-CdR-only cells. CFTR silencing with CFTR siRNA inversely increased cell growth preventing 5-Aza-CdR effects compared with scrambled siRNA. Proliferating cells were also detected by 5-ethynyl-2′-deoxyuridine (EdU); this compound is incorporated into newly synthesized DNA during the S phase of the cell cycle. While A253 cells were significantly labeled with EdU, 5-Aza-CdR-treated A253 cells showed little to no EdU uptake. Conversely, CFTR inhibition with CFTR_inh_-172 recovered EdU-labeled numbers to those similar to those receiving 5-Aza-CdR treatment only. CFTR siRNA also increased proliferation in the presence of 5-Aza-CdR as compared to scrambled siRNA (Figure 6B). These results demonstrate that 5-Aza-CdR could attenuate uncontrolled cell growth in A253 cancer cells, while downregulation or dysfunction of CFTR could induce aggressive cell growth, indicating CFTR can suppress uncontrolled proliferation of HNC.

Apoptotic cells were stained with PE and annexin-V and counted by flow cytometry. 5-Aza-CdR treatment increased A253 cell apoptosis compared with controls (Figure 6C). However, CFTR silencing with CFTR_inh_-172 (upper panel) or CFTR siRNA (lower panel) in 5-Aza-CdR-treated cells showed decreased numbers of apoptotic cells compared with 5-Aza-CdR-only treated cells or scrambled siRNA and 5-Aza-CdR-treated cells. The activation of caspases 3 and 7 was detected in apoptotic cells, indicating that 5-Aza-CdR treatment increased apoptosis, while apoptosis levels decreased following inhibition of CFTR (Figure 6D) These data suggest that CFTR is critically induced anti-proliferation and apoptosis in A253 cells.

### 3.7. Epigenetic Induction of CFTR Negatively Regulates Cell Motility and Invasion.

**Figure 7:**
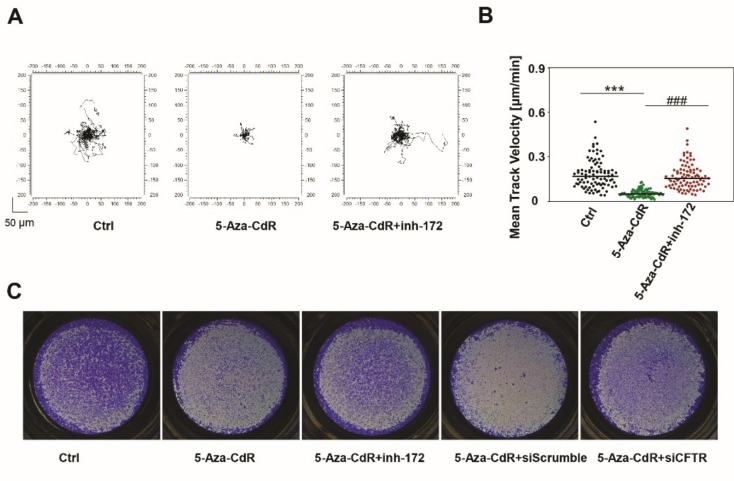
Epigenetic induction of CFTR regulates cell motility and invasion in A253 cells. (**A**) Representative 24 h time-lapse image from control (DMSO), 5-Aza-CdR-treated cells, and CFTR_inh_-172 and 5-Aza-CdR-treated cells showing 30 single cell motilities. A253 cells were aggressively motile, but 5-Aza-CdR treatment decreased this motility, and CFTR inhibition with CFTR_inh_-172 increased it. (**B**) Corresponding histograms quantifying motility parameters. Values are mean ± SD of the tracking of 90 cells (from three independent experiments; one-way ANOVA). (**C**) Matrigel invasion assay demonstrates that 5-Aza-CdR treatment largely mitigated invasion, and CFTR inhibition (CFTR_inh_-172, CFTR siRNA) increased the number of invading cells. Statistical significance was assessed using one-way ANOVA with Bonferroni’s test. Data are expressed as mean ± SD (*n* = 3; *** or ^###^, *p* < 0.001). * indicates statistical significance compared with control; # indicates significance compared with 5-Aza-CdR-treated cells.

Tumor cell motility underlies invasion and metastasis, as such, cell motility is a frequent target of cancer therapeutics [32,33,34]. Time lapse imaging was used to measure the motility of thirty cells every 30 min for 24 h (Figure 7A). Individual cell migration and distances of A253 cells were diverse and aggressive, and 5-Aza-CdR treatment was associated with significantly suppressed cell motility. The inhibition of CFTR with CFTR_inh_-172 in 5-Aza-CdR-treated A253 cells, however, significantly promoted cell motility to values close to those in controls. The graph in Figure 7B shows the velocity of ninety total cells, accounting for triplicated experiments.

A Matrigel invasion assay was performed to measure the invasiveness of cancer cells. DMSO-treated A253 cells highly invaded the Matrigel, while 5-Aza-CdR treatment was associated with fewer penetrating cells (Figure 7C). CFTR silencing with CFTR siRNA or CFTR_inh_-172 reversed the anti-invasive 5-Aza-CdR effects; numbers of invaded cells were increased in CFTR-silenced cells. These data indicate that CFTR is implicated in 5-Aza-CdR suppression of cell motility and invasion in HNC and suggest anti-invasion CFTR activity.

## 4. Discussion

CFTR is an indispensable ion channel for epithelial function and was recently discovered to participate in tumor progression or suppression depending on the type of tumor [35,36]. While CFTR upregulation has been observed in ovarian, cervical, gastric, and nasopharyngeal cancers, hypermethylation and/or downregulation of CFTR were reported in prostate, breast, colorectal, and bladder cancers, non-small cell lung carcinoma (NSCLC), and hepatocellular carcinoma (HCC) [37].

We first identified the downregulation and aberrant DNA methylation of CFTR in HNC, A253 cells, and human tissues. Emerging evidence indicates that cancer-associated hypermethylation is observed in many tumors that leads to transcriptional silencing of tumor suppressor genes [5]. Our study provides evidence that hypermethylation of CpG islands of CFTR is correlated with the loss of CFTR expression in HNC. Conversely, 5-Aza-CdR demethylated hypermethylated CpG islands in CFTR, and this was followed by restoration of CFTR expression. Notice that both upregulation of mRNA and protein and an increase in 8-Bromo-cAMP-sensitive chloride current was observed in 5-Aza-CdR-treated cells. No current was detected in control cells without 5-Aza-CdR, likely due to a lack of CFTR expression.

Likewise, our results confirmed that 5-Aza-CdR could potently demethylate aberrantly hypermethylated tumor suppressor genes, and these results recapitulated those of many other studies. Furthermore, the epigenetic drugs are susceptibility to aberrant methylated genes rather than normal cells [38]. The DNA methylation inhibitor known to function on dividing cells, so preferentially activate the genes which abnormally silenced in cancer, so nondividing normal cells are not affected by this drug [39]. On the other hand, importantly, optimal usage of 5-Aza-CdR is critical in clinical therapy, and low doses are sufficient to demethylate aberrant hypermethylated tumor suppressor genes as opposed to normal cells and high doses of 5-Aza-CdR increase cytotoxicity and lead to the demethylation in normal cells [11]. Accordingly, the optimal dose schedule of 5-Aza-CdR in each type of tumor must be individually titrated to each patient’s tolerance and response [40]. Besides, our data with 5-Aza-CdR treatment yielded an interesting pattern: despite CFTR hypermethylation in A253 HNC cells, the CpG islands of CFTR in 5-Aza-CdR-treated A253 cells became highly demethylated and this hypomethylation level was even greater than control cells (Figure 3). We also obtained consistent results in Figure 1 that the expression of CFTR was intact in HSG, but 5-Aza-CdR-treated A253 had slightly stronger expression of CFTR than HSG. These results indicate efficient demethylation by the epigenetic drug, 5-Aza-CdR and confirm negative correlation between methylation and mRNA expression. A recent study reported that although there is significant difference in methylation levels between cancerous and normal cells, CpG islands in normal cells are less likely unmethylated and show various methylation status depending on epigenetic, genomic, and functional nature, and also on the tissue types [41], but a complete understanding of how these factors affect CpG island methylation is yet lacking.

Cancer epigenetics, the study of epigenetic modifications to genes affecting cellular phenotypes of cancer cells by mechanisms other than changes to DNA sequence, has been actively studied within the past two decades and uncovered novel biomarkers and therapeutic targets for most type of cancers [42,43,44,45]. Even the molecular alterations causing diverse malignancy traits can be deeply influenced by epigenetic changes [42]. Epigenetic modification including histone modification, non-coding RNA regulation, and aberrant DNA methylation have been associated with tumor initiation, cancer progression, and metastasis [44]. To date, methylation is the most well-studied mechanism in epigenetic modification. Unlike normal and stable state of somatic cells, methylation is dynamic in embryogenesis and tumorigenesis [46]. DNA hypermethylation can be a useful diagnostic and prognostic tool, and DNA hypomethylation analysis is used for detecting cancer and managing the disease [3]. Aberrant DNA hypermethylation of CpG islands can be detected early on in carcinogenesis due to associations thereof with inflammation and aging, suggesting methylation-based approaches may reveal crucial markers for early diagnosis of HNC [47,48,49,50]. Though our study focused on methylation, future clinical studies might probe the underlying mechanisms of epigenetic networks among epigenetic mechanisms. While cancer epigenetics approaches have been successful and DNMT and HDAC inhibitors are clinically advanced and have been approved by the FDA, underlying mechanisms of epigenetic drugs and a deeper understanding of signaling processes could further enlighten epigenetic therapy.

We closely examined CFTR activity in cancer characteristics and potential signaling compared to other tumors. We found reduced proliferation after 5-Aza-CdR treatment, whereas CFTR inhibition in the presence of 5-Aza-CdR promoted cell growth, suggesting CFTR negatively regulates proliferation. Another study reported that a lack of CFTR promoted proliferation associated with an increase of extracellular ATP in breast cancers [51]. In prostate cancer, urokinase plasminogen activator (uPA) has been suggested as key signal for CFTR-induced anti-cancer responses to proliferation and invasion. CFTR was shown to upregulate microRNA-193b (miR-193b), a tumor suppressor, after which miR-193b suppressed uPA, frequently associated with malignant traits of cancer development [52] These results suggest that CFTR is responsible for growth restriction. We also observed that CFTR silencing decreased apoptosis rates, while CFTR induction by 5-Aza-CdR enhanced them. A previous report reported that CFTR increased apoptosis by increasing the pH and ceramide levels in the lung [53]. CFTR is also known to participate in cisplatin, a chemotherapy drug, induced apoptosis by regulating glutathione in cisplatin-sensitive A549 cell lines in NSCLC [54]. We demonstrated that CFTR inhibition aggressively increased cell motility and invasion and prevented the effects of 5-Aza-CdR. Of note, a previous study reported that downregulation of CFTR was associated with a proportional downregulation of E-cadherin and activation of NFκB/uPA, resulting in disruption of cell polarity, induction of epithelial-to-mesenchymal transition (EMT), and invasion in breast cancer [51]. In addition, CFTR was shown to inhibit invasion by suppressing NFκB in esophageal cancer cells [55]. Increasing evidence also points to direct or indirect regulation of cell-to-cell junctions by CFTR [56,57]. CFTR has been shown to colocalize and interact with AF-6/afadin, a cell junction protein, in the colon [58]. In intestinal cancer, CFTR has been proposed as a tumor suppressor gene that regulates other tumor-related genes [59].

CFTR tumor suppressor activity has been previously identified in several cancers including breast, prostate, NSCLC, and intestinal cancer [51,52,60]. CFTR deficiency is associated with reduced disease-free survival in human colorectal cancer [60], poor prognosis in human breast cancer [51], and poor survival rates in young patients with NSCLC [61]. These previous studies on CFTR in other cancers, lead to a hypothesis that CFTR might be deeply related to metastasis, poor prognosis, or even poor survival rates. Five-year survival rates for HNC patients with metastasis are extremely low, at about 10%, while rates for other HNC patients are about 50% [62]. The addressing of these challenges by early diagnosis of HNC and the definition of HNC metastasis indicators is urgently required. This study investigated the effect of CFTR on the initial step of the metastasis cascade (proliferation, anti-apoptosis, cell motility, and invasion) in HNC cells and further study may include identification of CFTR functions in HNC metastasis and EMT from both animal and clinical data. We consistently and extensively showed that CFTR induction by 5-Aza-CdR was associated with attenuation of aggressive cancer characteristics. Conversely, CFTR silencing counteracted the effects of 5-Aza-CdR treatment. Therefore, epigenetic drugs and CFTR inhibition might give good insight into developing promising therapeutic agents for head and neck cancer.

Taken together, our data show that the epigenetic modification of CFTR was associated with cancer activity in HNC. As we described earlier, the hypermethylation of tumor suppressor genes are key for early diagnosis and prognosis, leading to the increase of the survival rates. Therefore, we suggest that CFTR and its cascade signaling can be novel biomarkers for early diagnose and useful for prognostic studies for HNC.

Further study will be required to identify the underlying mechanisms of CFTR methylation in HNC. Studies have introduced a range of diverse signals for each different tumor and its microenvironment that may underlie the contradictory activity of CFTR depending on the type of cancer, but the underlying mechanisms are yet under investigation [35]. CFTR has nonetheless been associated with a broad array of proteins and the regulation of multiple signaling pathways and biological processes, suggesting current study on CFTR-associated signaling has just begun to scratch the surface [63]. In this study, we demonstrated tumor suppressor activity of CFTR in HNC and found several candidate genes potentially regulated by CFTR. A better understanding of these detailed CFTR signals in different tumors will open up new avenues for therapeutic research.

## 5. Conclusions

To the extent of our knowledge, this is the first report on aberrant epigenetic silencing and epigenetic modulation of CFTR in HNC. Our data indicate that CpG island hypermethylation of CFTR turns off its transcriptional expression. However, 5-Aza-CdR reversed this epigenetic modification and was associated with the rescue of functional CFTR expression. 5-Aza-CdR-induced CFTR recovery was also associated with apoptosis, anti-proliferation, low-cell motility, and anti-invasion. Our findings demonstrate that CFTR silencing may be associated with cancer progression in HNC and strongly suggest CFTR as a potential tumor suppressor and diagnostic and prognostic biomarker.

## Supplementary Materials

The following are available online at https://www.mdpi.com/2077-0383/9/3/734/s1, Figure S1: Downregulation and hypermethylation of CFTR in SGT; Figure S2: CFTR siRNA.
